# An Adaptationist Framework to Examine Intergroup Contact

**DOI:** 10.11621/pir.2022.0406

**Published:** 2022-12-30

**Authors:** John W. Berry, Dmitry Grigoryev

**Affiliations:** a HSE University, Moscow, Russia

**Keywords:** Intergroup contact, intercultural adaptation, psychological adaptation, personal wellbeing, multicultural ideology, prejudice, acculturation.

## Abstract

**Background:**

Many culturally-plural societies like Canada or Russia seek ways to manage their cultural diversity in order to promote harmony among coexisting groups. The social sciences have long viewed intergroup contact as a beneficial intervention to achieve such harmony.

**Objective:**

This paper proposes an adaptationist framework within which to explain how and why intergroup contact contributes to the positive and negative outcomes for individuals who live together in a plural society. We employed this framework in a case study that may serve as an example of the conceptualization and analysis of these issues in international research. Its structural framework included both positive and negative contact and the role of this contact in the distribution of intercultural and psychological adaptation among a large representative sample of the Canadian population.

**Design:**

We used a correlational design with a representative sample of Canadians from a survey carried out by Environics in 2019, which was stratified according to the most current population statistics. The total sample was 3,111 persons age 18 and over and included the largest racialised groups in the country.

**Results:**

Our main finding was that intergroup contact (both positive and negative) related to both psychological and intercultural adaptation. These findings have implications for improving intercultural relations, especially through the role of positive contact.

**Conclusion:**

The experience of negative contact (*e.g.,* discrimination) in the near term is an important factor in undermining both forms of adaptation. Nonetheless, while intergroup contact can bring both positive and negative experiences during intercultural interactions, it leads to mutual adaptation over time.

## Introduction

All societies are culturally diverse, including the Russian Federation. This poses the challenge of how we shall live together (Berry, 2017). Plural societies are made up of groups such as indigenous peoples, immigrants, different nationalities, and established ethnocultural groups, all of which differ in many respects, including their cultures, languages, and religions. At present, the main concern in these societies has come to focus on intergroup relations, particularly the phenomenon of racism (UNESCO, 2020).

Individuals develop their behaviours by adapting to their specific ecological and cultural contexts, a process which results in variations in behavioural repertoires across cultures (Berry et al., 2011). When individuals with these different repertoires meet in these diverse societies, they face the important issue of how they may adapt to each other and live successfully together (Berry et al., 2022). An ecocultural framework, developed by Berry (2018), proposes that variations in the development and display of features of peoples’ cultures and their individual behaviours can be accounted for by long-term adaptations to their ecological contexts and to their intercultural contact with members of other cultural groups. In addition to these cultural differences, one particularly salient difference between groups is the perceived physical differences in appearance (often thought of as “race”).

The mutual adaptation of these groups to living together has been studied for decades by researchers from many disciplines, including demography, economics, political science, sociology, and psychology. In the field of intercultural psychology, the overarching framework used in many of these studies has been rooted in the concept of acculturation (Sam & Berry, 2016), which has been defined as the cultural and behavioural changes resulting from intercultural contact (Berry, 2005). Various outcomes to acculturation have been conceptualized in different ways, in these studies, such as economic success, social engagement, cultural competence, and personal wellbeing. These different outcomes to living in these intercultural settings have been classified by Ward (2005) as psychological adaptation (“feeling well”) and sociocultural adaptation (“doing well”), to which Berry (2005) has added a third: intercultural adaptation (“relating well”).

The daily intercultural contacts of individuals in these culturally diverse societies creates a need for mutual adaptation. To meet this need, some societies have developed policies and practices to manage these relationships in order to achieve a positive intergroup climate. To deal with the diversity, Canada introduced a policy of multiculturalism (Canada, 1971). This policy has two main planks: promoting the value to a society of having diverse groups maintain their cultures over generations, and promoting contact among cultural groups. These two features (cultural diversity and contact among groups) provide a context that is ripe for the study of the effects of intergroup contact and its consequences (Berry, 1984; 2016). Since such contact among peoples of diverse backgrounds is a fundamental experience of daily life in all contemporary societies, the consequences of such contacts are a matter of great concern to policymakers, community leaders, and citizens, as well as social and behavioural scientists in many societies.

### Contact Hypothesis

One way of conceptualizing the consequences of intergroup contact has been formulated as the “contact hypothesis” (Allport, 1954). The core idea is that the more intergroup contact individuals have, then the more they will develop and express positive attitudes and behaviours towards individuals in the groups with which they are in contact. The contact hypothesis is one of the most enduring ideas in the field of intergroup relations (Christ & Kauff , 2019; Crisp & Turner, 2011; Dovidio et al., 2017; Kotzur et al., 2018; Pauluck et al., 2019; Pettigrew & Tropp, 2006). One characteristic of the contact hypothesis is the differentiation between amount of contact (how much and how often) and the quality of contact (friendly or hostile) (Bornmann, 2016; Nezlek & Schaafsma, 2010).

A good deal of research has been carried out to test the contact hypothesis internationally. In a large meta-analysis of this work, Pettigrew and Tropp (2006) examined hundreds of studies of the contact hypothesis, which were carried out in many countries and many diverse settings (in schools, at work, and in experiments). Their findings provided general support for the contact hypothesis: intergroup contact does generally relate negatively to prejudice in both dominant and non-dominant samples (see also Berry et al., 2022). That is, the results from the meta-analysis revealed that greater levels of intergroup contact are typically associated with a lower level of prejudice. In sum, the contact hypothesis proposes that under certain conditions, more intercultural contact will be associated with more mutual acceptance. Specifically, more contact will predict more positive intercultural adaptation.

Although contact is known to be related to intercultural adaptation, less is known about any relationship between contact and psychological adaptation (i.e., personal wellbeing). In this paper, we propose a relationship between individuals’ intercultural contacts and their psychological adaptation, as well as their intercultural adaptation. One basis for this possibility is the recent research finding that extensive social contacts promote the wellbeing of individuals. This relationship has been found for many kinds of social contacts, with many kinds of samples and in many societies (Jetten et al., 2015; Sønderlund et al., 2017).

Many studies have found such relationships between intercultural contacts and wellbeing, For example, in a study of first and second generation immigrants in Canada, Berry and Hou (2016, 2017) showed that social engagements with the larger society (termed “bridging social capital”) predicted higher self-esteem and mental health among immigrants and their descendants, and that contacts with their own ethnocultural group (termed “bonding social capital”) predicted higher life satisfaction. A three-year longitudinal study of refugees in the UK showed that intergroup contact at one point in time was associated with increased wellbeing at a later point in time but provided no reliable evidence for the reverse associations (Tip et al., 2018). In a study of Koreans who settled in New Zealand, Ward et al. (2020) examined the relationship between three aspects of normative multiculturalism (multicultural policies/practices; ideology; and contact) and wellbeing. They found that perceived multicultural policies and practices positively predicted subjective wellbeing, and multicultural ideology predicted wellbeing via a sense of belonging, but multicultural contact was not significantly related to wellbeing.

Some authors have raised questions about the reciprocity of contact and adaptive outcomes such as lower prejudice; that is, whether the intergroup contact is mutual, and is the cause and/or the effect of lower prejudice (Kalin & Berry, 1996; Nezlek & Schaafsma, 2010). In a longitudinal study with students, Binder et al. (2009) found that prior intergroup contact and liking led to subsequent intergroup contact, and that such contact promoted even greater acceptance of the other group, thus demonstrating a two-way relationship between contact and acceptance. We propose that these relationships constitute a behavioural syndrome: that is, the covariance of a set of psychological features involved in the process of adaptation to changing social and cultural conditions in plural societies.

The approach of coalitional psychology (Pietraszewski et al., 2014) considers the evolutionary core of intergroup relations to be a cognitive mechanism that evolved to detect coalitional alliances via the categorization of the social world into “Us” vs. “Them.” This mechanism is what ultimately predisposes humans to have a bias in favor of their ingroup and against the outgroup. For human beings, ethnic, cultural, or racial groups are simply one historically rooted type of coalition. This is because our long human history has shown this distinction to be an ecologically valid predictor of people’s social alliances and coalitional affiliations (Grigoryev et al., 2020). Adaptation to cultural diversity following contact takes place through the redefinition of concepts and the adjustment of the boundaries of ingroup and outgroup (“Us” vs. “Them”). This process includes both improving intergroup attitudes (intercultural adaptation) and reducing the effects of the stress of the heterogeneous environment on personal wellbeing (psychological adaptation).

### Discrimination as a Form of Negative Contact

In our view, discrimination may be conceptualized as a form of negative contact, one which is hostile as opposed to positive or friendly. Culturally and racially diverse neighbourhoods expose people to negative as well as positive intergroup contact; they go together. While positive contact is associated with more positive mutual attitudes (as documented above), negative contact increases prejudice, and limits intercultural adaptation (Barlow et al., 2012). As reviewed above, the negative psychological consequences of discrimination for both adults and youth of non-dominant peoples have also been well-documented world-wide for non-dominant peoples (Carter et al., 2019; Paradies, 2006; 2015). These studies showed that the experience of discrimination has a negative impact on people’s psychological adaptation.

While the effects of perceived discrimination on the adaptation of non-dominant peoples have been studied and documented, their effects on dominant groups are less well known (Leonardelli & Brewer, 2001). This group’s experience of discrimination may be seen as the result of laws that mandate employment equity requirements, such as quotas, for example. This may generate feelings of resentment (Yang, 2000), and lead them to develop higher levels of racism and lower acceptance of multiculturalism.

### The Present Study

Our research examined the contact hypothesis in a culturally pluralistic society society (Canada) within an adaptationist framework. It concentrated on the role that both positive contact and negative contact play in shaping the quality of intergroup relations and personal wellbeing. It supplemented the descriptive findings of the Environics report (2019) by using multivariate statistics and structural modeling to show how these variables related to each other, and provided a framework for explaining these relationships. In addition, this adaptationist framework broadens our understanding of the role of contact in intercultural relations (see Berry et al., 2022).

We focused on four main classes of phenomena within the adaptationist framework: (1) intergroup contacts among individuals; (2) the individual and group experience of discrimination; (3) intergroup attitudes; and (4) personal wellbeing. We asked the question: Does the quantity and quality of intergroup contact and the experience of discrimination (i.e., negative contact) impact individuals’ attitudes (their intercultural adaptation) and wellbeing (their psychological adaptation)? The adaptationist framework posits a basic situation in which individuals from two or more groups engage in direct contacts, leading to eventual adaptation by individuals. The core idea is that individuals and groups have adapted psychologically to each other’s presence over the course of history and continue to do so at the present time (see the ecocultural framework, Berry, 2018, for background to this approach).

Based on our adaptationist framework, we expected that:

(H1). Both positive contact and negative contact are two distinct aspects of intergroup contact, and are associated;
(H2). Intercultural adaptation and psychological adaptation are positively associated with each other;
(H3). Positive contact predicts higher levels of both intercultural and psychological adaptation, whereas negative contact shows the opposite.

## Method

### Sample

The Environics survey sampled the population of Canada (over 18 years of age) online in April and May of 2019. The total sample was 3,111 persons. The sample was stratified to ensure representation by province, age, and sex, according to the most current population statistical breakdown (2016 Census). It had oversamples of the largest racialised groups in the country: Blacks, Chinese, South Asians, and Indigenous Peoples.

### Measures

#### Contact

In order to assess the degree and quality of inter-racial contact, we used three measures from the Environics survey:

1. Contact frequency. “In your daily life, how much contact do you, personally, have with people who have a different racial background than your own?” Answers ranged from 1 = no contact at all to 5 = a lot of contact (M = 3.87, SD = 1.23).
2. Contact quality. “And, in general, would you say the interactions you have with people with a different racial background than yours is friendly or unfriendly?” Answers ranged from 1 = *very unfriendly* to 5 = *very friendly* (*M* = 4.28, *SD* = 1.01).
3. Number of friends. “Do you have friends from racial groups different from your own?” Answers were either 0 = no or 1 = yes; 81% of respondents answered “yes.”
4. Discrimination. We used two items to distinguish between discrimination against the group, and against an individual personally (for more detail, see Rafiqi & Thomsen, 2021). For group discrimination, “Thinking about people close to you who share your racial background, to what extent do you think their lives have been affected because of discrimination due to their race?” Answers ranged from 1 = not at all to 5 = to a great extent. For personal discrimination, “Now thinking about your own experience, have you ever personally experienced discrimination or been treated unfairly because of your race or ethnicity?” Answers ranged from 1 = never to 5 = regularly. The mean of this scale was 2.28, SD = 1.15. The scale reliability measured by the Spearman-Brown coefficient was .79.

#### Intercultural Adaptation

We have previously argued (Berry & Kalin, 2000) that the conditions for intercultural harmony in culturally-plural societies include among other phenomena: an acceptance of cultural diversity in the society (i.e., a positive multicultural ideology), and having positive attitudes toward specific other groups in the society. In the present study, this positive pattern was referred to as intercultural adaptation.

1. *Negative attitudes*. We use items from the index of modern racism that was developed by Environics to refer to people’s general attitude regarding four specific racial groups in Canadian society. Participants were asked to respond with respect to one of four racialised groups (Blacks, Chinese, South Asians, and Indigenous Peoples) selected randomly, but excluding members of their own. For each respondent the same group was used for all four questions. Responses ranged from 1 = *strongly disagree* to 5 = *strongly agree* (*M* = 2.64, *SD* = 0.70). A high score indicated a high level of racism. It was made up of six items, e.g.: “Over the past few years [group] have gotten more economically than they deserve.” This scale’s reliability as measured by the omega coefficient was .63, which is a sufficient value for such a large-scale survey with non-psychodiagnostic purposes (Nunnally, 1978).
2. Multicultural ideology. In addition to using this modern racism measure, we used one of the items (“Generally speaking, Canada would be a better place if ethnic and racial groups maintained their cultural identities”) to assess the concept of Multicultural Ideology (originally developed as a 10-item scale by Berry et al., 1977). This ideology refers to the degree to which individuals accept the extant and continuing cultural diversity of Canadian society. In the present study, this one item had the lowest factor loading on the Modern Racism Index scale used by Environics and hence shows some discriminant validity; we thus decided to remove it from their original Modern Racism scale, and use it as a stand-alone measure for Multicultural Ideology. Responses ranged from 1 = *strongly disagree* to 5 = *strongly agree* (M = 3.16, SD = 1.32).

#### Psychological Adaptation

As noted above, research has shown that personal wellbeing is usually supported by having extensive social contacts (Jetten et al., 2015). Hence, having more contacts, including intercultural ones, may well promote psychological adaptation. Personal wellbeing is often measured by such concepts as satisfaction with life and personal health (e.g., Berry & Hou, 2019).

1. Life satisfaction. Life Satisfaction was assessed using the question: “All things considered, how satisfied are you with your life as a whole these days” (from 1 = very dissatisfied to 10 = very satisfied, [M = 6.91, SD = 2.31]). This is the usual question employed in Canadian national surveys and has been widely used in the subjective wellbeing literature (e.g., Bonikowska et al., 2014).
2. Personal health. The other questions used to assess psychological adaptation were: “In general, would you say your mental health is excellent, very good, good, fair, or poor?” (mental health) and “In general, would you say your health is excellent, very good, good, fair or poor?” (physical health). These answers were coded from 1 = poor to 5 = excellent (M = 3.32, SD = 0.93). The items were derived from the RAND Corporation Short Form Survey. These measures have been used by Statistics Canada in national health surveys since 2000. Some literature supports the items’ construct validity (Canadian Institute for Health Information, 2009). This scale reliability measured by the Spearman-Brown coefficient was .66.

## Results

**Figure 1.**
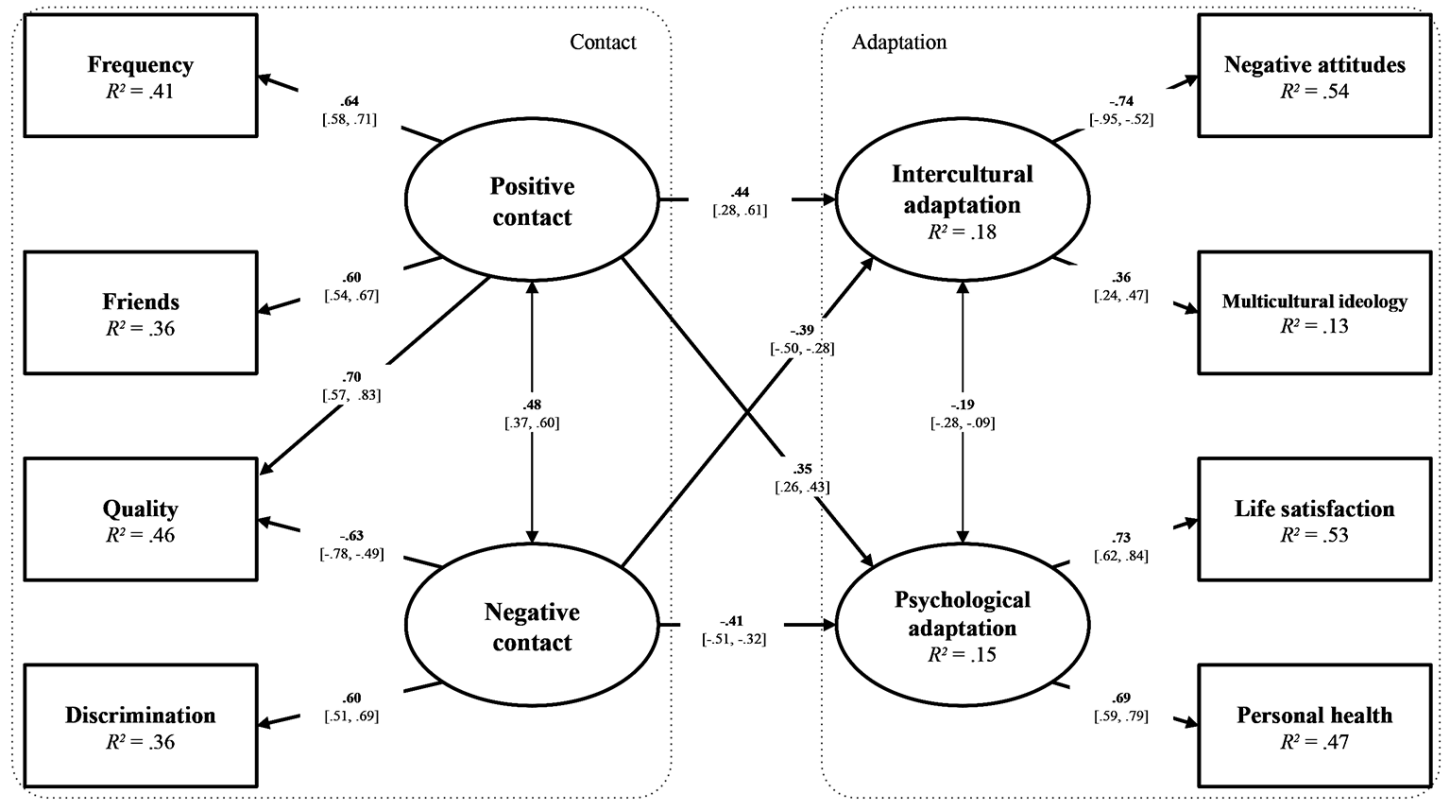
Structural model of relationships among contact, intercultural adaptation, and psychological adaptation

*Note. We used the estimator MLR and the FIML imputation method to deal with missings in the structural model, which showed standardized weighted estimates and their 95% CIs. Model fit was χ^2^(13, N = 3111) = 74.6, p < .001; CFI= .954; RMSEA [90% CI] = .039 [.033, .046]; SRMR = .026. All the coefficients were significant at p < .001*.

We present our results in the following sequence: correlations among focal variables in the sample (Table 1); multivariate linear (OLS) regressions predicting intercultural and psychological adaptation in the sample (Tables 2); and the structural model for relationships among the variables (Figure 1). For all of the analyses, we used the same sample weights as Environics.

**Table 1:** Weighted Bivariate Correlations of the Focal Variables

	1	2	3	4	5	6	7
Intercultural adaptation							
1. Negative attitudes	–						
2. Multicultural ideology	–.25*	–					
Psychological adaptation							
3. Life satisfaction	.01	.06*	–				
4. Personal health	.09*	.05*	.49*	–			
Contact							
5. Frequency	–.06*	.04*	.04	.04*	–		
6. Friends	–.13*	.13*	.06*	.02	.34*	–	
7. Quality	–.18*	.14*	.18*	.17*	.19*	.22*	–
8. Discrimination	.10*	.08*	–.11*	–.06*	.23*	.17*	–.18*

Note. * = p < .05

**Table 2:** Standardized Weighted Estimates and Their 95% CIs Predicting Intercultural Adaptation (Negative Attitudes and Multicultural Ideology) and Psychological Adaptation (Life Satisfaction and Personal Health)

	Intercultural adaptation		Psychological adaptation	
	Negative des attitu-	Multicultural ideology	Life satisfaction	Personal health
Socio–demographic variables				
Sex 1 = (male) 0 = female;	.16 [.11, .22]*	–.01 [–.11, .09]	–.19 [–.36, –.02]*	.06 [–.01, .13]
Age	.02 [–.01, .05]	–.25 [–.30, –.20]*	.38 [.29, .46]*	.12 [.09, .16]*
Education	–.07 [–.10, –.04]*	.04 [–.02, .09]	.15 [.06, .24]*	.02 [–.02, .05]
Income	.04 [.01, .07]*	–.05 [–.10, .01]	.38 [.29, .47]*	.11 [.07, .15]*
Contact				
Frequency	–.01 [–.04, .02]	–.09 [–.14, –.03]*	.04 [–.05, .13]	.02 [–.02, .06]
Friends	–.18 [–.26, –.11]*	.30 [.16, .44]*	.20 [–.03, .43]	–.04 [–.14, .05]
Quality	–.09 [–.12, –.06]*	.20 [.15, .26]*	.27 [.18, .36]*	.14 [.10, .18]*
Discrimination	.07 [.04, .10]*	.09 [.04, .14]*	–.12 [–.21, –.03]*	.01 [–.04, .04]
	*R^2^* = .07	*R^2^* = .07	*R^2^* = .10	*R^2^* = .06
	*F*(8, 2525) = 24.4, *p* < .001	*F*(8, 2525) = 25.2, *p* < .001	*F*(8, 2525) = 35.1, *p* < .001	*F*(8, 2525) = 21.2, *p* < .001

Note. * = p < .05

### Preliminary Analysis

#### Correlations

1. Contact Measures. The three positive contact variables (frequency, quality, and friends) were positively correlated (Table *1*) among themselves (range from +.19 to +.34). This allowed their later use as a single combined contact variable in the structural model. Two of the contact measures (quality and discrimination) were correlated (–.18) and were used to create a negative contact variable.
2. Adaptation Measures. The two measures of intercultural adaptation (negative attitudes and multicultural ideology) were negatively and significantly correlated as expected (–.25). The correlation between the two measures of psychological adaptation (life satisfaction and personal health) was also significant as expected (+.49). Life satisfaction was positively associated with multicultural ideology only (+.06), whereas personal health was positively associated with both negative attitudes and multicultural ideology (at +.09 and +.05, respectively).

#### Regressions

Contact quality had a beneficial effect on all four adaptation variables in the sample (Table 2). Contact frequency had a negative association with multicultural ideology. Contact with friends had a beneficial effect on the two intercultural adaptation variables, but not on the psychological ones. Discrimination had variable effects on adaptation: it undermined life satisfaction in the sample and showed opposite patterns for both forms of intercultural adaptation.

### Structural Equation Model (SEM)

To present an overall picture of how all these variables are related, we created Figure 1. It shows a structural model with a combined positive contact variable (made up of three constituent variables of frequency, quality, and friends) and a combined negative contact variable (made up of two constituent variables of quality of contact and discrimination). These were used to predict the latent variables of intercultural adaptation (made up of negative attitudes and multicultural ideology) and psychological adaptation (made up of life satisfaction and personal health). This structural model initially showed a good model fit and did not require any post hoc modification. Thus, the SEM model with the fit, which meets the “gold standard” (i.e., CFI> .950, SRMR < .050; see e.g., Hu & Bentler, 1999), showed that our interpretation of covariances between the focal variables, based on our adaptationist framework, did not contradict the data.

The core features of interest are the relationships between the combined contact variable and the two adaptation latent variables. First, our main finding was that positive contact predicted both positive intercultural adaptation and psychological adaptation, while negative contact predicted the opposite (H3). Second, the two main predictor variables (positive and negative contact) were positively related (H1), whereas, unexpectedly, the two adaptation variables were negatively related (H2).

## Discussion

We used our adaptationist framework to examine the role of positive and negative contact in intercultural and psychological adaptation in a case study with the Canadian population. In general, the findings and interpretations of the present paper corresponded with those of the Environics report. However, the present analyses shed more light on the question ‘what goes with what’ by using multivariate statistics and putting the findings into an explanatory adaptationist framework.

### Contact and Adaptation

Both the regression models and the structural model revealed that positive contact (especially a high quality of contact) supported and promoted both forms of adaptation in the Canadian population. This can mean not only that majority group members could possibly aid the wellbeing of minority groups by seeking contact with them (Tip et al., 2018), but that contact with minority group members could aid majority group members as well. This finding of the mutual benefits of contact corresponds with the international findings of Berry et al. (2022) in their examination of mutual intercultural relations. That is, intercultural contact is not a zero-sum game, but a win-win opportunity for all groups.

This benefit of contact for both forms of adaptation, means that “relating well” and “feeling well” are linked to positive contact in the same way. The only other study to examine a relationship between intergroup contact and the wellbeing of members of ethnocultural groups (Ward et al., 2020), did not find any relationship. However, they did find that multicultural ideology positively predicted wellbeing. In the present study, multicultural ideology was also positively related to life satisfaction and personal health.

The positive association between multicultural ideology and negative contact (i.e., discrimination) needs explanation. This positive relationship may mean that respondents react to discrimination by increased favoritism for their ingroup (higher multicultural ideology and less negative attitudes; see e.g., Verkuyten, 2007). Taking into account the cross-sectional design of this study (which lacks a time perspective), a possible interpretation is that the more that individuals endorse multicultural ideology and the less they endorse negative attitudes, the lower will be the level of discrimination against them in the future. Thus, this relationship may be a functional and adaptive response of individuals to discrimination in the present in order to avoid these experiences in the future.

Of further interest in these relationships was the finding that they hold for both dominant and non-dominant groups in the contact. A mutuality or reciprocity in intercultural relations corresponds with the main contention and finding of the project on mutual intercultural relations (Berry, 2017). This study often found common views about intercultural relations by both dominant and non-dominant groups in the 17 societies (and the more than 40 groups) studied. This reciprocity showed that when one group likes the other, the other reciprocates this positive affect. The finding indicates that there is no trade-off or “zero-sum” character to contact: everyone benefits (as found by Berry et al., 2022).

### Contact

The correlations among the three indicators of positive contact (in Table 1) are all significant and positive, indicating that they go together: more frequent contact is associated with higher quality contact and having more friends in other groups. Of course, the number of friends available outside one’s own group varies as a demographic factor between groups: in most neighbourhoods more members of the non-racialised group are available to racialised persons than the other way around. Earlier studies in Canada (Kalin, 1996; Kalin & Berry, 1982) showed that judgments of “familiarity” with a specific ethnocultural group were related to their actual presence in a neighbourhood. Moreover, the larger the group’s population, the more positive were the attitudes towards them. The findings in current studies (almost 50 years later) indicate that the social ecology of the community may play a role in how much intergroup contact there is, and how well each group accepts the other.

Positive and negative contact were positively related because contact is a function of social interaction. Indeed, neighbourhood diversity has been shown to be positively associated with both positive and negative intergroup encounters (see Prati et al., 2022). Hence, more contact means more both positive and negative contact, but normally the weight of positive contact is higher, and ultimately this leads to adaptation in a population.

### Adaptation Relationships

Of particular interest in this study was whether there were any consistent relationships between the two forms of adaptation. The positive contribution of contact to both forms of adaptation in both kinds of groups noted above suggested that there could be some kind of consistency: since more contact promoted better adaptation, the two forms of adaptation might also be positively related. But no; they were negatively related.

This negative association between the two forms of adaptation can be understood in the context of the positive correlations between personal health, and both negative attitudes and multicultural ideology, as evident in the results. Negative racial bias among people has been found to have a negative effect on their psychological distress (Samson, 2018) and in non-self-report measures of personal health (Lee et al., 2015; Leitner et al., 2016). However, the present study used a self-report measure of personal health. There was possibly a confounding factor here: perhaps it was due to some common optimistic bias among some respondents that led to overrating both their personal health and the situation with respect to racism in Canada.

### Implications

#### Theoretical Implications

A recent cross-country study (Shira, 2020) showed that the historical level of cultural heterogeneity (over the past 500 years) was associated with lower levels of prejudice among the population, while the current level of cultural heterogeneity in the country has the opposite relationship. Other authors argue that populations tend to react negatively to threats to their homogeneity in the short term, while in the long run, these negative results are offset by people getting to know the beneficial effects of intergroup contact, which mitigates the initial negative effects (Ramos et al., 2019). Thus, the possible mechanism behind the contact hypothesis may be that people over time simply adapt to a culturally heterogeneous environment, in keeping with our adaptationist perspective.

The experience of intergroup interactions over time can enrich the cognitive and behavioral repertoire of individuals, thereby adapting individuals to a culturally diverse context. For example, intergroup contact leads to changes in perceptive processing at the neural level (Farmer et al., 2020; for extended reading, see Amodio & Cikara, 2021). Also, stereotypes change in the process of socio-cognitive adaptation to a new cultural environment (Stanciu et al., 2019) and to more heterogeneous contexts (Bai et al., 2020); they also reduce perceptions of threat (McKenna et al., 2018). A common beneficial result of intergroup contact is termed cognitive liberalization, which suggests the presence of generalized cognitive flexibility beyond the realm of intergroup relations (Hodson et al., 2018; also see Verkuyten et al., 2022). Intergroup contact also leads to affective changes (e.g., reductions in group-based anxiety and increases in empathy). These affective changes are even stronger than changes that involve enhanced knowledge of the other group (Pettigrew & Tropp, 2006). They can indicate reduced chronic stress from the impact of this new heterogeneous environment as well.

Meleady et al. (2019) presented a taxonomy of transfer effects that explained the generalization effects as distinct outcomes of the contact process. This taxonomy included three types of transfer effect: (1) primary transfer effect (i.e., when intergroup contact enables generalized improvements in attitudes toward outgroups as a whole); (2) secondary transfer effect (i.e., when intergroup contact can enable generalized improvements in attitudes toward other, non-contacted outgroups); and (3) tertiary transfer effect (i.e., when intergroup contact impacts more general cognitive processes outside the intergroup context; this is termed cognitive liberalization). All of these effects can be considered consequences of evolved person-environment fit mechanisms in our adaptationist framework.

The structural model presented in this study is an example of how to use our adaptationist framework. Dovidio et al. (2017) noted that some important lacunae remain after 20 years of research progress, and suggested that future research might explore the health consequences of intergroup contact. We propose that our adaptationist framework can be an especially helpful perspective.

#### Practical Implications

Our findings show support for the contact hypothesis, using the individual measures of contact (in the correlations) and the combined measure (in the structural models). In addition to showing support for the usual relationship between positive contact and mutual acceptance (intercultural adaptation), we also showed that contact is positively related to psychological adaptation. That is, contact not only promotes more harmonious relations between groups, but also higher levels of psychological wellbeing.

## Conclusions

Despite lingering questions, we conclude that the contact hypothesis has largely been supported in the present study, with respect to both intercultural and psychological outcomes. We also conclude that the experience of negative contact (e.g., discrimination) in the near term is an important factor in undermining both forms of adaptation. Nonetheless, while intergroup contact can bring both positive and negative experiences during intercultural interactions, it leads to mutual adaptation over time. We suggest using such an adaptationist framework to carry out future research into intergroup relations in order to further develop the approach.

## Limitations and Further Research

There are some critiques that contact measures do not distinguish between different outgroups and do not differentiate the various types of contacts people have (e.g., personal socializing vs. work contact; Nezlek & Schaafsma, 2010). In the present study, we have dealt with the second point by differentiating between having friends, and the frequency and quality of contact. However, we have not dealt with the first point. Furthermore, in a complex multicultural society, contact among members of different minority groups has received relatively little attention (van Oudenhoven & Ward, 2013).

While our findings are representative and generalizable for the Canadian population, future research with other representative samples could explore the relationships for the specific groups that make up the multicultural Canadian society. Moreover, future research could be carried out in other plural societies (e.g., Russia) using this adaptationist framework. This research could employ the same replication strategy used in the MIRIPS study (Berry, 2017; Berry et al., 2022) to search for possible universal relationships between intergroup contact and psychological and intercultural adaptations.

Another limitation concerns using previously collected data to operationalise the focal variables. The items available in the Environics survey are not the best measures to test all components of our adaptationist framework. For example, the survey asked respondents to provide retrospective accounts of their intergroup contact over unspecified periods of time, which may serve as a bias in assessing these variables. Further analyses into the structure of the data presented in this paper may also be carried out after a second survey to examine how these complex relationships develop over time, despite the likely changes in the levels of contact and mutual adaptation.
